# Response to Vasavda et al, “Delayed diagnosis of a generalized annular rash following immunosuppression”: A letter to the editor

**DOI:** 10.1016/j.jdcr.2026.02.050

**Published:** 2026-03-20

**Authors:** Will Swansson, Anthony Moussa, Kristof Wing, Laura Scardamaglia

**Affiliations:** a Department of Dermatology, The Royal Melbourne Hospital, Parkville, Victoria, Australia; b Department of General Medicine, The Royal Melbourne Hospital, Parkville, Victoria, Australia; c Department of Medicine, The University of Melbourne, Melbourne, Victoria, Australia; d Department of Dermatology, Western Health, Melbourne, Victoria, Australia; e Department of Dermatology, The Royal Children’s Hospital, Melbourne, Victoria, Australia

**Keywords:** crusted scabies, hyperkeratosis, immunosuppression, Janus kinase inhibitors, Norwegian scabies, prurigo nodularis, upadacitinib

*To the Editor*: We read with interest a recent report by Vasavda et al of a 73-year-old man who developed crusted scabies while on the selective JAK1 inhibitor, upadacitinib.^1^

We have encountered a similar presentation in an 85-year-old woman treated with upadacitinib for refractory prurigo nodularis. Three months after treatment initiation, she developed widespread thick, fissured plaques over the bilateral elbows, forehead, left upper eyelid, thighs, and bilateral feet. Skin scrapings and biopsies confirmed the diagnosis of Grade 3 crusted scabies. Upadacitinib was permanently discontinued and her condition improved following intensive antiscabietic therapy.

Together, our cases emphasize that nonclassical scabies can present with various atypical morphologies, which may result in delayed diagnosis or misdiagnosis. Although severe pruritus is a hallmark of classical scabies, in crusted scabies it can be variable. Furthermore, the potent antipruritic effect of Janus kinase (JAK) 1 inhibitors may further reduce clinical suspicion.

Although immunosuppression is a recognized risk factor for crusted scabies, reports in association with JAK1 inhibitors remain scarce.^1^^,^^2^ JAK1-dependent cytokine signaling is central to CD4+ T-cell–mediated defense against *Sarcoptes scabiei*,^3^ providing biological plausibility that JAK1 inhibition may attenuate antiscabietic immunity.

Crusted scabies is associated with high morbidity and transmissibility, particularly in hospital and aged care settings, and swift identification is essential.^4^ It is therefore important to consider crusted scabies in the differential diagnosis for new-onset hyperkeratotic, crusted, or atypical scaly eruptions, including in the setting of JAK inhibitor therapy.

## Conflicts of interest

None disclosed.
